# Environmental Growth Conditions of *Trichoderma* spp. Affects Indole Acetic Acid Derivatives, Volatile Organic Compounds, and Plant Growth Promotion

**DOI:** 10.3389/fpls.2017.00102

**Published:** 2017-02-09

**Authors:** Maria F. Nieto-Jacobo, Johanna M. Steyaert, Fatima B. Salazar-Badillo, Dianne Vi Nguyen, Michael Rostás, Mark Braithwaite, Jorge T. De Souza, Juan F. Jimenez-Bremont, Mana Ohkura, Alison Stewart, Artemio Mendoza-Mendoza

**Affiliations:** ^1^Bio-Protection Research Centre, Lincoln UniversityLincoln, New Zealand; ^2^Institute for Scientific and Technological Research of San Luis PotosiSan Luis Potosí, Mexico; ^3^Department of Phytopathology, Federal University of LavrasLavras, Brazil; ^4^School of Plant Sciences, University of ArizonaTucson, AZ, USA; ^5^ScionRotorua, New Zealand

**Keywords:** *Trichoderma*, auxins, 3-indole-acetic acid, plant growth promotion, volatile organic compounds, 6-PP

## Abstract

*Trichoderma* species are soil-borne filamentous fungi widely utilized for their many plant health benefits, such as conferring improved growth, disease resistance and abiotic stress tolerance to their hosts. Many *Trichoderma* species are able to produce the auxin phytohormone indole-3-acetic acid (IAA), and its production has been suggested to promote root growth. Here we show that the production of IAA is strain dependent and diverse external stimuli are associated with its production. In *in vitro* assays, *Arabidopsis* primary root length was negatively affected by the interaction with some *Trichoderma* strains. In soil experiments, a continuum effect on plant growth was shown and this was also strain dependent. In plate assays, some strains of *Trichoderma* spp. inhibited the expression of the auxin reporter gene DR5 in *Arabidopsis* primary roots but not secondary roots. When *Trichoderma* spp. and *A. thaliana* were physically separated, enhancement of both shoot and root biomass, increased root production and chlorophyll content were observed, which strongly suggested that volatile production by the fungus influenced the parameters analyzed. *Trichoderma* strains *T. virens* Gv29.8, *T. atroviride* IMI206040, *T*. sp. “*atroviride B”* LU132, and *T. asperellum* LU1370 were demonstrated to promote plant growth through volatile production. However, contrasting differences were observed with LU1370 which had a negative effect on plant growth in soil but a positive effect in plate assays. Altogether our results suggest that the mechanisms and molecules involved in plant growth promotion by *Trichoderma* spp. are multivariable and are affected by the environmental conditions.

## Introduction

Plant-microbe interactions in the rhizosphere are key determinants of plant health, productivity and soil fertility (Souza et al., 2015). Plant roots synthesize metabolites that are recognized by microorganisms, which in response, produce signals that initiate microbial colonization (Berg, 2009). Plant roots also secrete sucrose as a food source to support colonization by the microorganisms (Druzhinina et al., 2011; Vargas et al., 2011).

The mechanism by which microorganisms promote plant growth has previously been studied for both bacteria (Crozier et al., 1988; Ahmad et al., 2005; Idris et al., 2007) and fungi (Contreras-Cornejo et al., 2009; Salazar-Badillo et al., 2015). It has been suggested that over 80% of rhizosphere bacteria synthesize indole-3-acetic acid (IAA) (Patten and Glick, 1996), the plant hormone auxin that controls many aspects of plant growth and development (Grossmann, 2010). The ability to synthesize indole-3-acetic acid (IAA) is an attribute that many microorganisms possess, including both plant growth-promoters and some plant pathogens (Duca et al., 2014). IAA synthesized by plant root-associated microorganisms can interfere with plant development by disturbing the auxin balance in plants. For example, IAA synthesis can modify root architecture, resulting in increased root mass, and consequently, an increased area suitable for microbial colonization and a larger root system for nutrient uptake by the plant (Spaepen et al., 2007; Berg, 2009; Contreras-Cornejo et al., 2009).

*Trichoderma* species are common soil-borne filamentous fungi, with some strains capable of establishing beneficial relationships with plants (Harman et al., 2004; Druzhinina et al., 2011; Contreras-Cornejo et al., 2014). Direct plant growth promotion (Rando and Verstrepen, 2007; Richards et al., 2010; Jablonka, 2013) is one of many mechanisms in which *Trichoderma* spp. enhance plant health, however, the molecular mechanism that underlies this is still unclear. Contreras-Cornejo et al. (2009) suggested that *Trichoderma* spp. induce growth promotion by a fungal auxin-dependent mechanism. Using *in vitro* bioassay, they showed that *Trichoderma virens* Gv29.8 and *T. atroviride* IMI206040 can synthesize IAA (and some of its derivatives) and have been demonstrated to increase the production of *Arabidopsis* lateral roots (Contreras-Cornejo et al., 2009). However, the correlation between IAA synthesis and plant growth promotion of plants in soil-based systems is less convincing. Hoyos-Carvajal et al. (2009a) showed that many *Trichoderma* strains are capable of synthesizing IAA but only a few can promote plant growth.

Other microbially produced molecules known to be important in promoting plant growth are the microbial Volatile Organic Compounds (mVOCs), which have been recognized as key players in plant growth promotion (Vespermann et al., 2007; Minerdi et al., 2011; Zhi-Lin et al., 2012; Paul and Park, 2013; D'Alessandro et al., 2014; Garnica-Vergara et al., 2015; Kanchiswamy et al., 2015; Lee et al., 2015, 2016; Salazar-Badillo et al., 2015). mVOCs are low-molecular weight lipophilic compounds that can easily evaporate at room temperature and pressure, and suggested to play a role in long distance communication between organisms. mVOCs belong to different chemical classes, including mono- and sesquiterpenes, alcohols, ketones, lactones, esters, thioalcohols, thioesters and cyclohexenes (Schenkel et al., 2015). Sesquiterpenes from ectomycorrhizal fungi have been shown to induce lateral root formation in diverse plants such as *Populus* and *A. thaliana* (Ditengou et al., 2015). 6-pentyl-2H-pyran-2-one (6-PP) is a compound detected in diverse *Trichoderma* species, including *T. atroviride* IMI206040 (Reithner et al., 2005; Stoppacher et al., 2010), *T. asperellum* (Wickel et al., 2013; Kottb et al., 2015), *T. viride* (Collins and Halim, 1972), *T. harzianum* (Claydon et al., 1987), *T. koningii* (Simon et al., 1988), *T. citrinoviride*, and *T. hamatum* (Jeleń et al., 2014), and its production by *T. atroviride* has been shown to induce lateral root formation in *A. thaliana* (Garnica-Vergara et al., 2015). Intriguingly not all *Trichoderma* species synthesize 6-PP (Atanasova et al., 2013) yet most are able to induce plant growth promotion (Kottb et al., 2015) suggesting the correlation is not strong as observed recently by Lee et al. (2016).

The aim of this study is to analyse the effect of diverse *Trichoderma* strains on plant growth promotion and identify the signals that may be involved. Three well-studied *Trichoderma* model strains and seven *Trichoderma* strains previously screened for their ability to promote root growth in willow (*Salix* x *matsudana*) (Braithwaite et al., 2009) and *Impatiens walleriana* (Clouston et al., 2010) were selected for this study using *A. thaliana* as a host.

## Materials and methods

### Fungal material

The New Zealand *Trichoderma* strains used in this study were isolated from different locations and chosen for their ability to induce root development in cuttings of commercial ornamental plant species. These strains were sourced from the Microbial Biocontrol Culture Collection (MBCC, Bio-Protection Research Centre, Lincoln University) and represent a range of species as well as strains with previously known biocontrol and growth promotion activity (Supplementary Table 1S). *Trichoderma reesei* QM6a (Simmons, ATCC® 13631) was provided by The American Type Culture Collection (ATCC). *Trichoderma atroviride* IMI206040 and *T. virens* Gv29.8 were kindly provided by Alfredo Herrera-Estrella (Langebio, Mexico) and Charles Kenerley (Texas A&M, USA), respectively. All *Trichoderma* strains in the MBCC were stored in 25% glycerol at −80°C and were previously identified using morphology and five different genetic markers (*tef1*α*, ACLA1, Calm1, LAS1, and RPB2*) under separate research initiatives (Hoyos-Carvajal et al., 2009b; Braithwaite et al., 2016). Strains LU132, LU660 and LU668 were closely related to *T. atroviride* and were defined as *Trichoderma* sp. “*atroviride* B” (Braithwaite et al., 2016).

For all experiments, fungal inoculum consisted of conidia harvested from 7 d old PDA cultures grown at 25°C under a 12/12 light/dark photoperiod. The resulting conidial suspension was filtered through two layers of Miracloth (Merck Millipore) and adjusted to the concentration required for each experiment.

### Plant material

All mutants and transgenic lines are derived from parental *Arabidopsis thaliana* ecotype Col-0. DR5::GUS (Ulmasov et al., 1997). DR5rev::GFP (Friml et al., 2003), PIN1::PIN1-GFP (Benková et al., 2003), PIN2::PIN2-GFP (Xu and Scheres, 2005), PIN4::PIN4-GFP (Blilou et al., 2005), PIN7::PIN7-GFP (Blilou et al., 2005) and PIN3::PIN3-GFP (Zádníková et al., 2010) transgenic lines were previously described and are listed in Supplementary Table 2S.

### Influence of *Trichoderma* on *Arabidopsis* growth in sterile soil

*Trichoderma* strains with plant growth promotion potential were evaluated for their ability to promote growth of the model plant *Arabidopsis* in sterile soil. *Arabidopsis thaliana* (Col-0) seeds were surface sterilized in 95% (v/v) ethanol for 5 min and 20% (v/v) bleach for 7 min, washed five times with sterile water, and kept in 2 mL sterile water at 4°C for 2 d to break dormancy and synchronize germination. Seeds were then incubated for 5 d at 25°C under a 16 h light: 8 h dark (16L: 8D) day/night cycle on standard MS medium: 0.2 X MS medium (Murashige and Skoog basal salt mixture, catalog M5524, Sigma-Aldrich) containing 1.0% (w/v) agar (Sigma), then supplemented with 0.6% sucrose and adjusted to pH 7.0 (Lopez-Bucio, personal communication) before being poured into Petri dishes. The resulting seedlings were then transferred to gamma irradiated sterile soil (chemical composition is described in Mendoza-Mendoza et al., 2015) which was previously inoculated with 1 × 10^6^ spores per g soil of *Trichoderma* spp. (*T. trixiae* LU297, *T*. sp. “*atroviride* B” LU660, *T*. sp. “*atroviride* B” LU668, *Trichoderma* sp. nov LU753, *T*. sp. “*novaeharzianum*” LU1328, *T. asperellum* LU1370, *T. atroviride* IMI206040). The plants were assessed 4 weeks after planting for survival and the total fresh weight was measured. The experimental design was a complete randomized block with 7 treatments and 1 negative control with 4 replicates, a total of 32 plots. Each plot consisted of 4 pots (50 mm diameter, 50 mm deep) containing a single *Arabidopsis* plant. The experiment was repeated twice with similar results.

### Influence of *Trichoderma* on *Arabidopsis* root architecture on agar plates

Enhanced growth in the presence of *Trichoderma* is likely due to changes in the plant root architecture. To investigate how the *Trichoderma* strains influence root structure, strains were evaluated in an *Arabidopsis* agar plate bioassay (Contreras-Cornejo et al., 2009). Ten sterilized/stratified seeds were placed on MS agar (section Influence of *Trichoderma* on *Arabidopsis* Growth in Sterile Soil) in a row 1.5 cm from the edge of the Petri dish. The Petri dishes were sealed with plastic film to prevent cross-contamination and moisture loss and placed on their edge at an angle of approximately 65° to allow root growth down along the agar surface and upward shoot growth. The seedlings were pre-incubated for 5 d at 25°C under a 16L: 8D day/night cycle (Morange, 2002) and then 1 × 10^6^ *Trichoderma* spores (strains mentioned in section Influence of *Trichoderma* on *Arabidopsis* Growth in Sterile Soil, and *T*. sp. “atroviride” B LU132; *T. virens* Gv29.8 and *T. reesei* QM6a) in a 5 μL aliquot of water were inoculated 5 cm below the growing root tips. Petri dishes were re-sealed with plastic film and incubated for a further 5 d under the same conditions mentioned above. Control plates were inoculated with water only. At the end of the experiment, the length of each primary root was measured and the number of lateral roots counted. The root density was then calculated as a function of lateral root number divided by the primary root length (Contreras-Cornejo et al., 2009). Each *Trichoderma* strain was assayed in four independent experiments using 10 replicate Petri dishes.

### Effect of *Trichoderma* on *Arabidopsis* auxin accumulation and transport using transgenic *Arabidopsis* lines

Regulation of plant auxins has been linked to root growth promotion by microorganisms. To investigate if the presence of *Trichoderma* influences auxin accumulation in the plant, confrontation assays were done using an *A. thaliana* transgenic line with the auxin responsive DR5 promoter fused to the β-glucuronidase gene (*gus*) as described by Ulmasov et al. (1997). Assays were conducted as described in section Influence of *Trichoderma* on *Arabidopsis* Root Architecture on Agar Plates except *A. thaliana* DR5::GUS was selected as the plant line. After 5 d interaction with *Trichoderma* spp. (*T. atroviride* IMI206040, *T. reesei* QM6a, *T*. sp. “*atroviride* B” LU668, *T. virens* Gv29.8, *T*. sp. “*atroviride* B” LU132, *T*. sp. “*atroviride* B” LU660, *T. asperellum* LU1370) DR5::GUS seedlings were removed and incubated at 37°C overnight in GUS staining solution (50 mM sodium phosphate buffer, pH 7.0, 0.1% Triton X-100, 1.5 mM potassium ferricyanide, 1.5 mM potassium ferrocyanide, and 1.5 mg/mL X-Gluc). After staining, the samples were cleared by incubation with 7% NaOH for 20 min and 60 min with 100% ethanol followed by 60 min hydration with serial ethanol dilutions 70, 60, 40, 20, and 10%. Then the seedlings were incubated for 30 min with 25% glycerol and overnight with 50% glycerol at room temperature. Seedlings were visualized using an Olympus BX51 compound microscope. Images were captured using an Olympus DP70 digital camera system and processed with the software Cell^F^ (Olympus). The experiment was repeated once with similar results using 10 replicate plants for each repeat.

For the localization of the different auxin transporters, confrontation assays were performed as described above using *T. virens* Gv29.8 for inoculum and multiple *Arabidopsis* transgenic lines. These lines correspond to the marker for auxins DR5::GFP, and different auxin transporters fused to the green fluorescent protein: PIN1::PIN1-GFP, PIN2::PIN2-GFP, PIN4::PIN4-GFP, PIN7::PIN7-GFP and PIN3::PIN3-GFP (Supplementary Table 2S). After 5 d interaction, seedlings were removed from the interaction plate and directly mounted on a slide for observation on an Olympus BX51 compound microscope. Images were captured using an Olympus DP70 digital camera system and processed with the software Cell^F^ (Olympus). The experiment was repeated once using 10 replicate plants each time.

### TLC analysis of indole derivatives from *Trichoderma*

Thin layer chromatography (TLC) was used to further investigate the role of auxins in *Trichoderma* growth promotion. IAA and its derivatives were isolated and identified from different *Trichoderma* strains (*T*. sp. “*atroviride* B” LU660, *T*. sp. “*atroviride* B” LU668, *T*. sp. “*novaeharzianum*” LU1328, *T. asperellum* LU1370, *T*. sp. “*atroviride* B” LU132, *T. atroviride* IMI206040, *T. virens* Gv29.8, and *T. reesei* QM6a). *Trichoderma* spp. spores were inoculated at 1 × 10^6^ spores/mL in 100 mL of 0.2 X MS (pH 7.0) in 500 mL flasks and incubated shaking (120 rpm) for 48 h at 25°C. Mycelia were collected by filtration on sterile filter paper (Whatman 3MM) and equally divided before inoculated in either 0.2 × MS (pH 7.0) with or without 10 mM L-Tryptophan (Trp). Flasks were incubated shaking (120 rpm) for an additional 24 and 48 h at 25°C. The supernatant from each culture was then filtered to remove the fungal material. The supernatants were adjusted to pH 3.0 with HCl and indole derivatives extracted from 30 mL of supernatant mixed with 15 mL ethyl acetate, shaking at 200 rpm for 3 h in darkness. The organic phase was recovered and evaporated in a CentriVap Centrifugal Vacuum Concentrator (Labconco). The dry pellet was dissolved in 50 μL of ethyl acetate and 5 μL of each sample was loaded onto a TLC silica gel 60. The TLC silica gel was sprayed evenly with van Urk and Salkowski reagents (1:3) (Ehmann, 1977) and developed by incubation at 90°C for 7 min. The indole derivatives were separated for 45 min using n-hexane:ethyl acetate:isopropanol:glacial acetic acid (40:20:5:1, v/v) as mobile phase (Chung et al., 2003). Indole derivative compounds were identified against commercial standard compounds (L-Tryptophan, Tryptamine hydrochloride, 3-indole acetonitrile, DL-indole-3-lactic acid, indole-3-acetaldehyde, and indole-3-acetic acid) from Sigma-Aldrich; 25 μg of each indole standard was loaded per lane. To compare IAA production under different nutrient conditions, a second experiment was done using PDB instead of MS medium and strains *T*. sp. “*atroviride* B” LU132, *T. reesei* QM6a, *T. atroviride* IMI206040, and *T. virens* GV29.8 were assayed. Both assays were repeated once.

### Effect of *Trichoderma* indole derivatives on *Arabidopsis* auxin accumulation

The activity of *Trichoderma* indole-derivatives on *Arabidopsis* auxin accumulation was assessed using an *A. thaliana* transgenic line with the auxin responsive DR5 promoter fused to β-glucuronidase gene (*gus*) as described by Ulmasov et al. (1997). DR5::GUS *A. thaliana* seeds, were surface sterilized and stratified as described in section Influence of *Trichoderma* on *Arabidopsis* Growth in Sterile Soil and individually germinated for 6 d in wells of a 96-microtiter plate containing 100 μL of standard MS broth at 25°C under 16 h light/8 h dark settings. MS broth was then replaced with 100 μL fresh standard MS broth containing 10 μL of ethyl acetate containing the dissolved pellet from supernatant extracts from *T*. sp. “*atroviride* B” LU132, *T. reesei* QM6a, *T. atroviride* IMI206040, or *T. virens* GV29.8 grown with or without tryptophan for 48 h (section Effect of *Trichoderma* on *Arabidopsis* Auxin Accumulation and Transport Using Transgenic *Arabidopsis* Lines) and incubated for an additional 24 h. Each treatment was allocated 16 wells. The control for the solvent was ethyl acetate, 10 μg/mL of commercial indole-3-acetic acid (sodium salt, Sigma Aldrich) was used as positive control, and distilled water as the negative control. The seedlings were incubated for 24 h at 25°C under 16 h light/8 h dark settings. Seedlings were removed and GUS staining was conducted as described above in section Effect of *Trichoderma* on *Arabidopsis* Auxin Accumulation and Transport Using Transgenic *Arabidopsis* Lines. Ten replicates per treatment were assessed and the entire experiment was repeated once.

### Effect of *Trichoderma* volatile organic compounds on plant growth and chlorophyll content

The effect of *Trichoderma* VOCs on plant growth promotion and chlorophyll content was assessed using a split-plate system. Five seeds of *A. thaliana* (Col-0) were surface sterilized and stratified as described in section Influence of *Trichoderma* on *Arabidopsis* Growth in Sterile Soil and then placed onto one side of a 90 mm diameter double compartment Petri plate (Labserv) containing Standard MS medium. Plates were sealed with plastic film and placed on their edge at an angle of approximately 65° and incubated at 22°C under 16 h light/8 h dark settings. After 7 d, 5 μL of a *Trichoderma* spore suspension (containing 1 × 10^6^ spores) was inoculated into the opposite compartment then the plates were re-sealed and incubated for additional 7 d. The *Trichoderma* strains used for this experiment were *T. atroviride* IMI206040, *T. virens* Gv29.8, *T*. sp. “*atroviride* B” LU132, *T. reesei* QM6a, and *T. asperellum* LU1370. Fresh weight of shoots, roots and total biomass (roots + shoots) per plant were measured. Shoots or roots of 10 plants were pooled and biomass was measured (*n* = 7, corresponding to 70 plants). The weight was divided by the number of plants to express the weight per plant. Relative chlorophyll content was quantified from 10 leaves per treatment. Chlorophyll content from the third leaf was determined using a SPAD 502 Plus Chlorophyll Meter. The SPAD values were converted to chlorophyll using the formula described by Ling et al. (2011) (nmol chlorophyll/cm2 = 0.0105 × 2 + 0.4119x + 0.3810). The experiment was carried out twice with 10 plants measured each time.

### Headspace analysis of *Trichoderma* VOCs

The VOC profiles of *T. atroviride* IMI206040, *T*. sp. “*atroviride* B” LU132, *T. virens* Gv29.8 and *T*. *asperellum* LU1370 were analyzed using a Shimadzu GCMS-QP2010 (Shimadzu Corporation, Japan) gas chromatograph - mass spectrometer fitted with a Restek Rxi-5 ms fused silica capillary column (30.0 m × 0.25 mm i.d. × 0.25 μm, Bellefonte, PA, USA) and an auto-sampler for solid phase micro-extraction (SPME). Fungal cultures were grown in Petri dishes on standard MS medium at 25°C and 12 h light/12 h darkness for 4 d. Four mycelial plugs (5 mm diameter) were punched out with a sterile cork borer from the outer growing zone of each *Trichoderma* colony and placed in an amber glass headspace vial (20 mL) sealed with a screw cap containing a blue PTFE/silicone septum (Sigma-Aldrich, Australia). The vials were incubated at 25°C for 24 h before sampling of the headspace VOCs was conducted according to Stoppacher et al. (2010). An SPME fiber coated with 65 μm polydimethylsiloxane/divinylbenzene was used to extract the fungal VOCs from the headspace vial for 30 min without agitation. After injection, the compounds were desorbed for 2 min in a split/splitless injector at 250°C. The oven temperature was held at 40°C for 2 min, then raised to 200°C at 10°C min^−1^ and 260°C at 25 min^−1^ and then held at this temperature for 5 min. Helium was used as carrier gas at a constant flux of 1 mL min^−1^. Compounds were identified by matching their mass spectra and linear retention indices using GCMS solution v. 2.72 (Shimadzu Corporation, Japan) software with NIST 11 and Wiley 10 mass spectrum libraries and by using the software MassFinder4 with a specialized terpenoids library (Hochmuth Scientific Software, Hamburg, Germany). The background of PDA plates without the fungus was extracted analysing the volatiles of four agar medium plugs. Three biological replicates were analyzed for each strain.

### Statistical analyses

**Figure 1:**
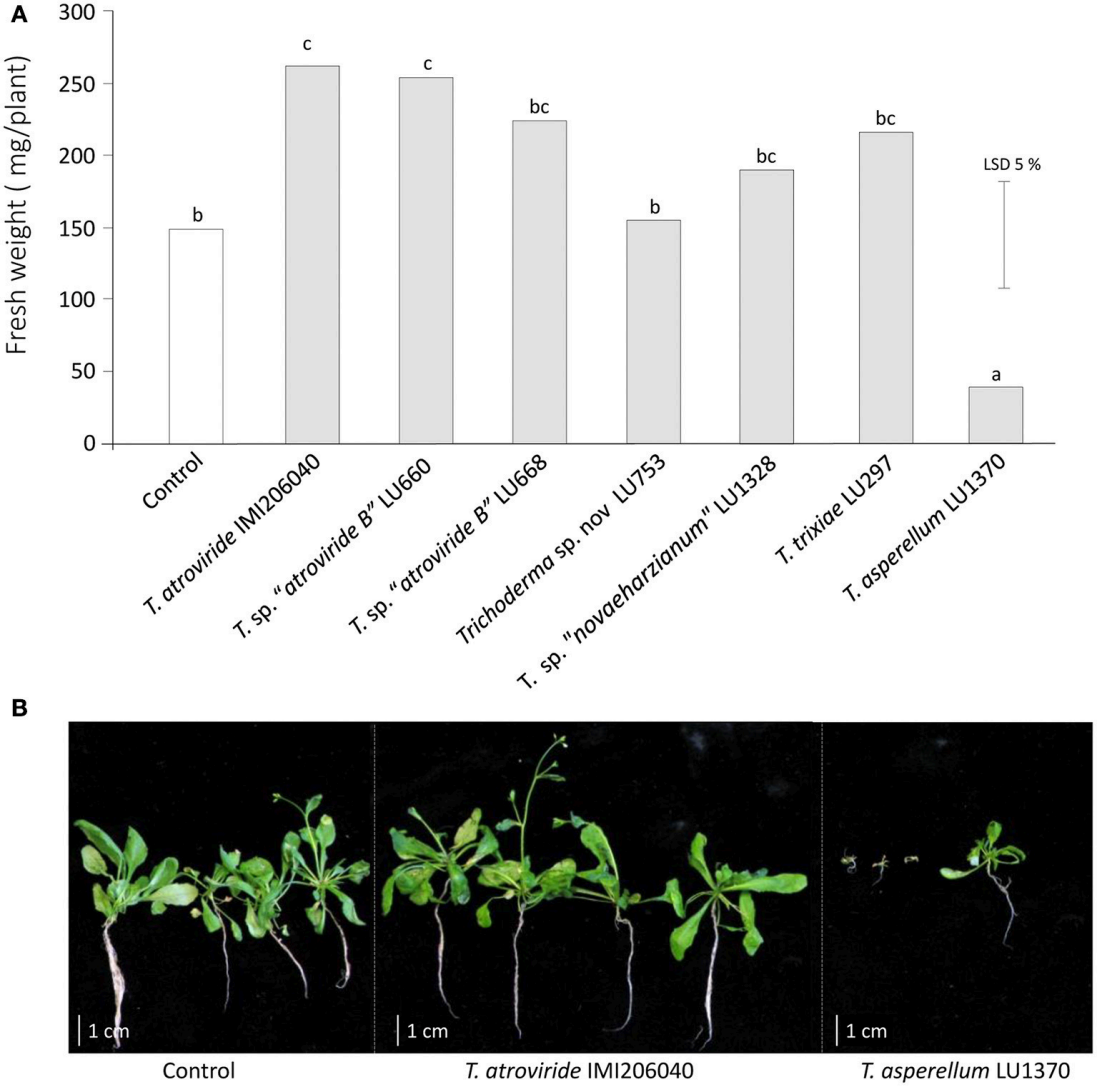
**Influence of ***Trichoderma*** inoculation on the fresh weight of ***Arabidopsis thaliana*** plants grown in sterile soil. (A)** Fresh weight of *A. thaliana* plants after 4 weeks interaction with *Trichoderma*. **(B)** Morphology of plant seedlings in co-culture with *T. atroviride* IMI206040 or *T. asperellum* LU1370.

**Figure 2:**
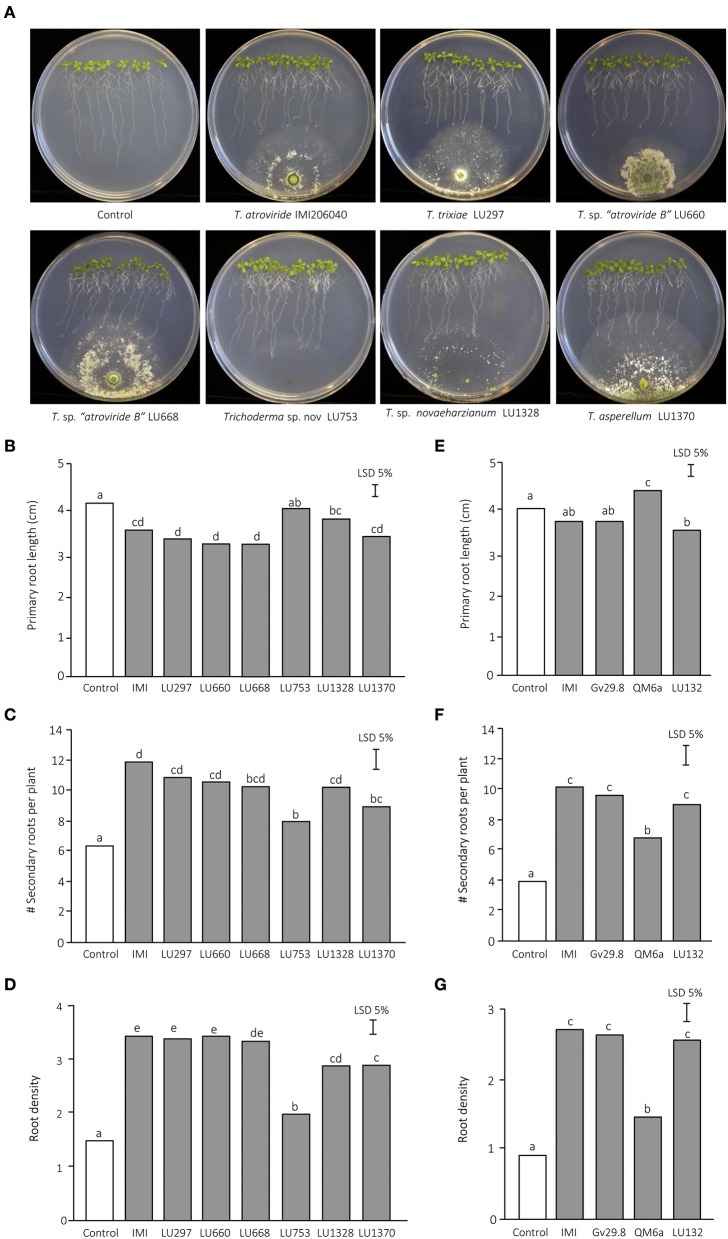
**Effect of ***Trichoderma*** on ***Arabidopsis thaliana*** root architecture. The results from two independent experiments are presented. (A)** *A. thaliana* seedlings after 5 d growth in the presence of *Trichoderma*. **(B)** Experiment 1—primary root length. **(C)** Experiment 1—number of emerged lateral roots per plant. **(D)** Experiment 1—root density. **(E)** Experiment 2—primary root length. **(F)** Experiment 2—number of emerged lateral roots per plant. **(G)** Experiment 2—root density.

Data analyses from fresh weight of *Arabidopsis* plants grown in sterile soil (Figure 1), effect of *Trichoderma* on *A. thaliana* root architecture (Figure 2), and the effect of *Trichoderma* volatile organic compounds on *Arabidopsis* plant biomass using a split-plate assay (**Figure 7**) were analyzed by Analysis of Variance (ANOVA) using the statistical software GenStat Version 18. Differences between treatment means were compared by the unrestricted least significant difference (LSD) analysis at *P* < 0.05.

## Results

### *Trichoderma* enhances and diminishes *Arabidopsis* growth in soil in a species/strain dependent manner

Seven *Trichoderma* strains, chosen for their ability to induce growth promotion in commercial plants, and the reference strain *T. atroviride* IMI206040, were tested for their ability to promote growth of *A. thaliana* in sterile soil. After 4 weeks of interaction, three outcomes were evident: an increase in plant biomass, a decrease in biomass, and no effect at all when compared to plants grown in un-inoculated soil. *Trichoderma atroviride* IMI206040 and *T*. sp. “*atroviride* B” LU660 increased *Arabidopsis* fresh biomass by 72% and 73% respectively, whereas *T. asperellum* LU1370 significantly inhibited *Arabidopsis* growth as measured by a reduction of up to 74% (*P* < 0.05) in biomass with respect to the plants grew in non-inoculated soil. *Trichoderma* sp. “*atroviride* B” LU668, *T. “novaeharzianum”* LU1328, *T. trixiae* LU297, and *Trichoderma* spp. LU753 had no significant effect on plant growth (Figure 1).

### *Trichoderma* spp. alter root architecture *in vitro*

The influence of *Trichoderma* inoculation on root architecture was investigated in more detail on MS agar. In the absence of *Trichoderma* spp., *A. thaliana* produced long primary roots with few secondary roots and consequently exhibited low root density (Figure 2). When the plates were inoculated with *Trichoderma*, the primary root length decreased by 13 to 25% and the number of secondary roots increased by 64 to 90%, which in turn resulted in a massive increase in root density (Figures 2B–D). To corroborate if the reduction of primary root length was a common phenomenon during the *Trichoderma* interaction, additional strains belonging to three different species were added to our analyses: *T*. *virens* Gv29.8, which has previously analyzed for their ability to induce *A. thaliana* secondary roots on MS medium (Contreras-Cornejo et al., 2009), *T*. sp. “*atroviride* B” LU132, a commercial biocontrol strain with plant growth promotion capability (Maag et al., 2014), and *T. reesei* QM6a, a commercial strain used for its capacity to secrete large amounts of cellulases and hemicellulases (Peterson and Nevalainen, 2012; Mukherjee et al., 2013; Strakowska et al., 2014) (Figures 2E–G). Two of these strains, *T*. sp. “*atroviride* B” LU132 and *T. virens* Gv29.8, induced a reduction in primary root length and an increase in the number of secondary roots, while *T. reesei* QM6a did not induce significant changes with respect to the control (Figures 2E–G). Interestingly, *Trichoderma* spp. LU753 did not significantly alter root architecture on MS plates even though it was capable of increasing root biomass in willow cuttings (Braithwaite et al., 2009). In trials on ornamental cuttings conducted on commercial nurseries, LU753 did not promote root biomass when applied alone but was effective when applied as part of an effective mixture which included two other *Trichoderma* strains (M. Braithwaite, pers. comm.) and *T*. sp. “*atroviride* B” LU660 promoted lateral root growth on *A. thaliana* in the plate bioassays (Figures 2B–D) but had no effect on root promotion in the willow screening trial.

A reddening of the leaves was observed for the majority of the *Trichoderma* treatments indicating strong anthocyanin production. Two strains (*T. sp. “novaeharzianum”* LU1328 and *T. reesei* QM6a) did not induce anthocyanin production (Supplementary Figure 1S).

### *Trichoderma* spp. differentially regulate auxins distribution in apical roots

**Figure 3:**
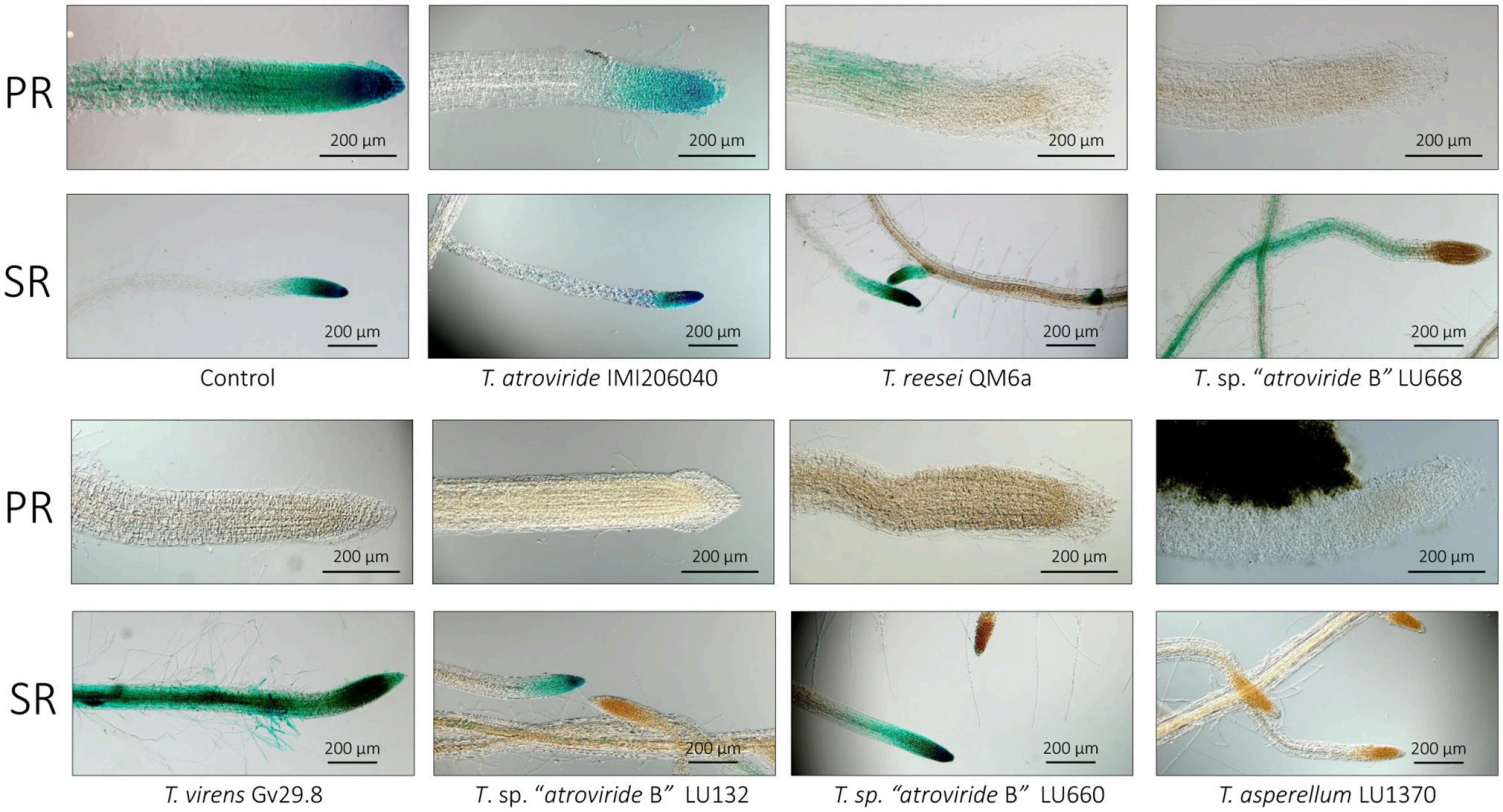
**Histochemical analysis of GUS activity driven by the DR5 promoter in the ***Arabidopsis*** DR5::GUS line after 5 d growth in the presence of ***Trichoderma*****. DR5::GUS expression levels in the Primary root (PR) and Secondary root (SR) tips. Scale bar, 100 μm.

The influence of *Trichoderma* spp. in the induction of auxins was assessed using DR5::GUS plant seedlings and *Trichoderma* strains on MS plates. DR5: GUS activity was profoundly visible in the primary and secondary root tips in the control seedlings (Figure 3). Unlike the control, GUS was weakly expressed in the primary root tip when *A. thaliana* DR5::GUS line interacted with *T*. *atroviride* IMI206040 and *T. reesei* QM6a. No GUS activity was observed in the primary roots with the remaining strains (Figure 3, PR). When secondary roots from *A. thaliana* DR5::GUS were analyzed, greater activity was observed in seedlings interacting with *T. virens* Gv29.8, *T*. sp. “*atroviride* B” LU668 and LU660, in comparison to the control and other *Trichoderma* strains (Figure 3, SR). Unexpectedly, *T. asperellum* LU1370 completely induced an inhibition of DR5 expression in secondary roots (Figure 3) but induced lateral root promotion on plates (Figure 2).

### Auxin PIN transporters are misallocated or non-expressed in *A. thaliana* during the interaction with *Trichoderma virens*

**Figure 4:**
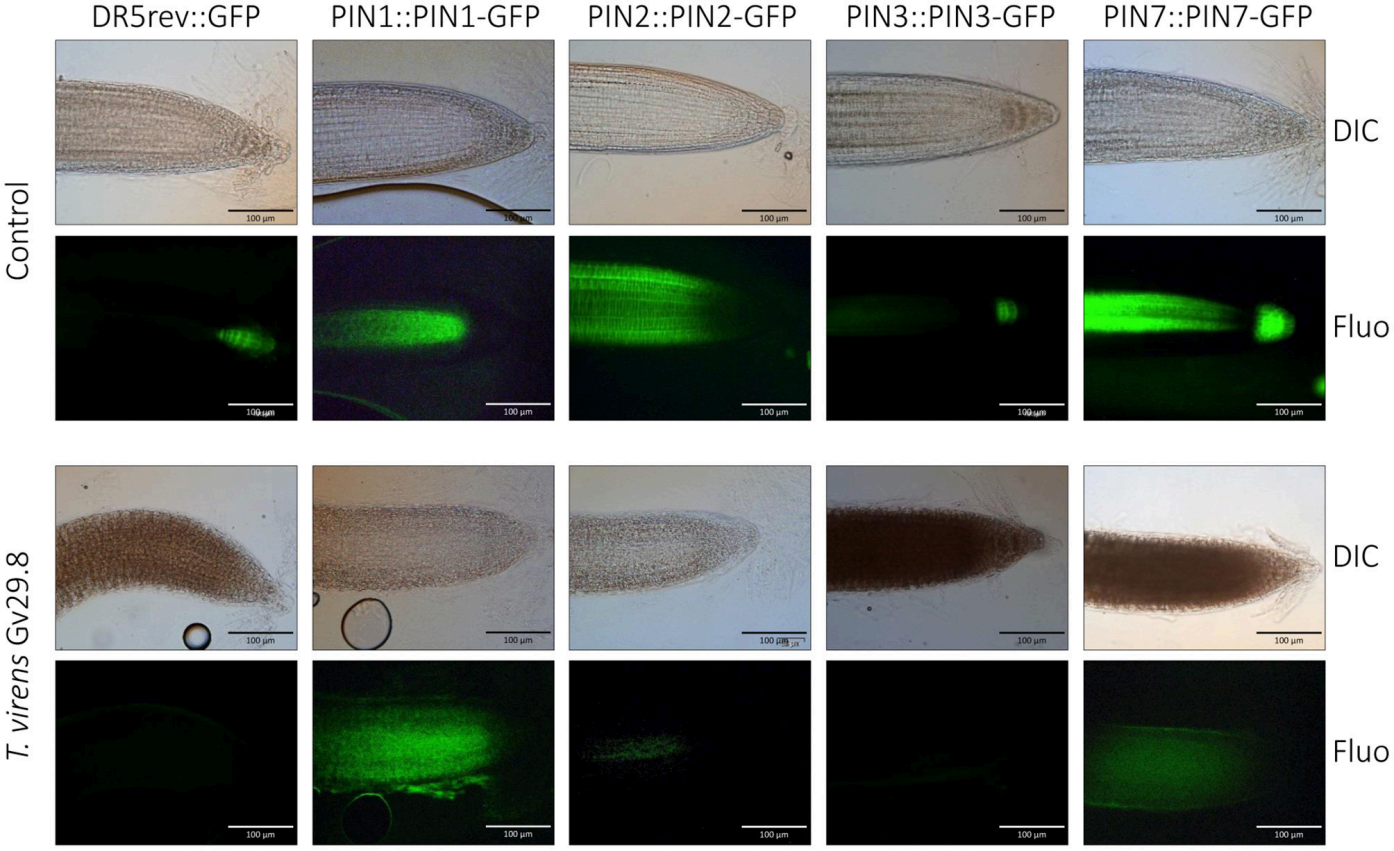
**GFP expression in ***Arabidopsis*** DR5REV::GFP and PIN transporter lines after 5 d growth in the presence ***of Trichoderma virens*** Gv29.8**. DIC = differential interference contrast. Fluo = fluorescence microscopy.

Although auxin is synthesized in the plant apices of shoots and roots, it is transported throughout the plant via the phloem, forming concentration gradients and accumulating in different tissues (Friml et al., 2003). PIN proteins operate as efflux carriers for auxin localization (Petrásek et al., 2006). These proteins are polarly localized in plants, including *A. thaliana* (Chen et al., 1998; Luschnig et al., 1998; Müller et al., 1998; Shin et al., 2005). To assess if polarity localization of PIN proteins is altered in *A. thaliana* by the interaction with *T. virens* Gv29.8, reporter lines of *A. thaliana* containing different PIN proteins fused to GFP were used. Expression of GFP modulated by DR5 was inhibited by the presence of *T. virens*, in contrast to the control where the typical localized GFP signal was observed (Figure 4). When the localization of PIN transporters in the primary roots (PIN1::PIN1:GFP, PIN2::PIN2:GFP, PIN3::PIN3:GFP, and PIN7::PIN7:GFP) were analyzed on MS medium, specific localization occurred in the different lines; however in the presence of the fungus, expression of PIN2 and PIN3 was completely inhibited and a diffuse localization of PIN1 and PIN7 was observed (Figure 4).

### Production of indole derivatives from *Trichoderma* spp. is highly variable among strains

**Figure 5:**
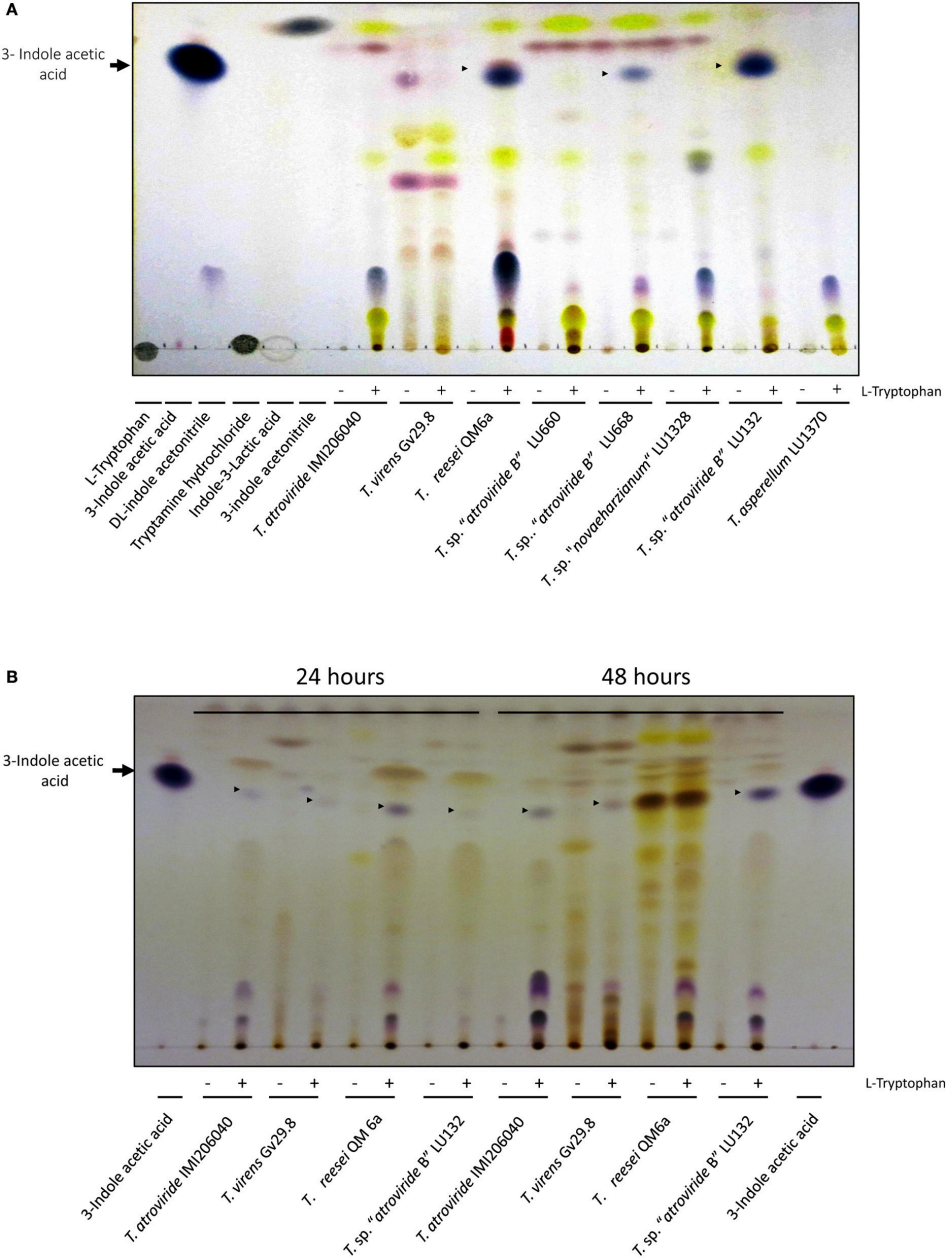
**Indoles derivatives recovered from supernatants of ***Trichoderma*** strains incubated with (+) or without (−) 10 mM of L-Tryptophan. (A)** Basal medium was MS. The standards used for identification of indole derivatives are indicated in the first 6 lanes. Samples incubated for 48 h with or without tryptophan are presented. **(B)** Basal medium was PDB. IAA was used as a standard in the first and last lane.

Changes in growth promotion activity by *Trichoderma* species on *Arabidopsis* plantlets may depend on their capacity to produce auxins. Therefore as a first step to investigate the role of auxins indole derivatives from *Trichoderma* were extracted and separated on TLC plates. On standard MS medium two *T*. sp. “*atroviride* B” strains (LU668 and LU132), *T. reesei* QM6a and *T. sp. “novaeharzianum”* LU1328 synthesized a compound that migrated to the same Rf as the commercial 3-indole acetic acid (IAA) (Figure 5). Interestingly, four strains did not produce IAA on MS standard medium (*T. atroviride* IMI206040, *T. virens* Gv29.8, *T*. sp. “*atroviride* B” LU660 and *T. asperellum* LU1370). In the absence of Trp, no production of indole derivatives was observed, with the exception of *T. virens* which produced a compound that migrated to the same Rf as IAA but with a different color; this was present but less evident when Trp was added (Figure 5A). Additionally *T. virens* synthesized additional compounds in standard MS independently of the presence of Trp.

Four strains from the first experiment were used to assess the IAA production in PDB: *T*. *atroviride* IMI206040, *T. virens* Gv29.8, *T*. sp. “*atroviride* B” LU132, and *T. reesei* QM6a. All strains were able to synthesize a molecule that migrated to the same Rf as commercial IAA (Figures 5A,B).

### Indole derivatives from *Trichoderma* supernatants and auxin accumulation in *A. thaliana*

**Figure 6:**
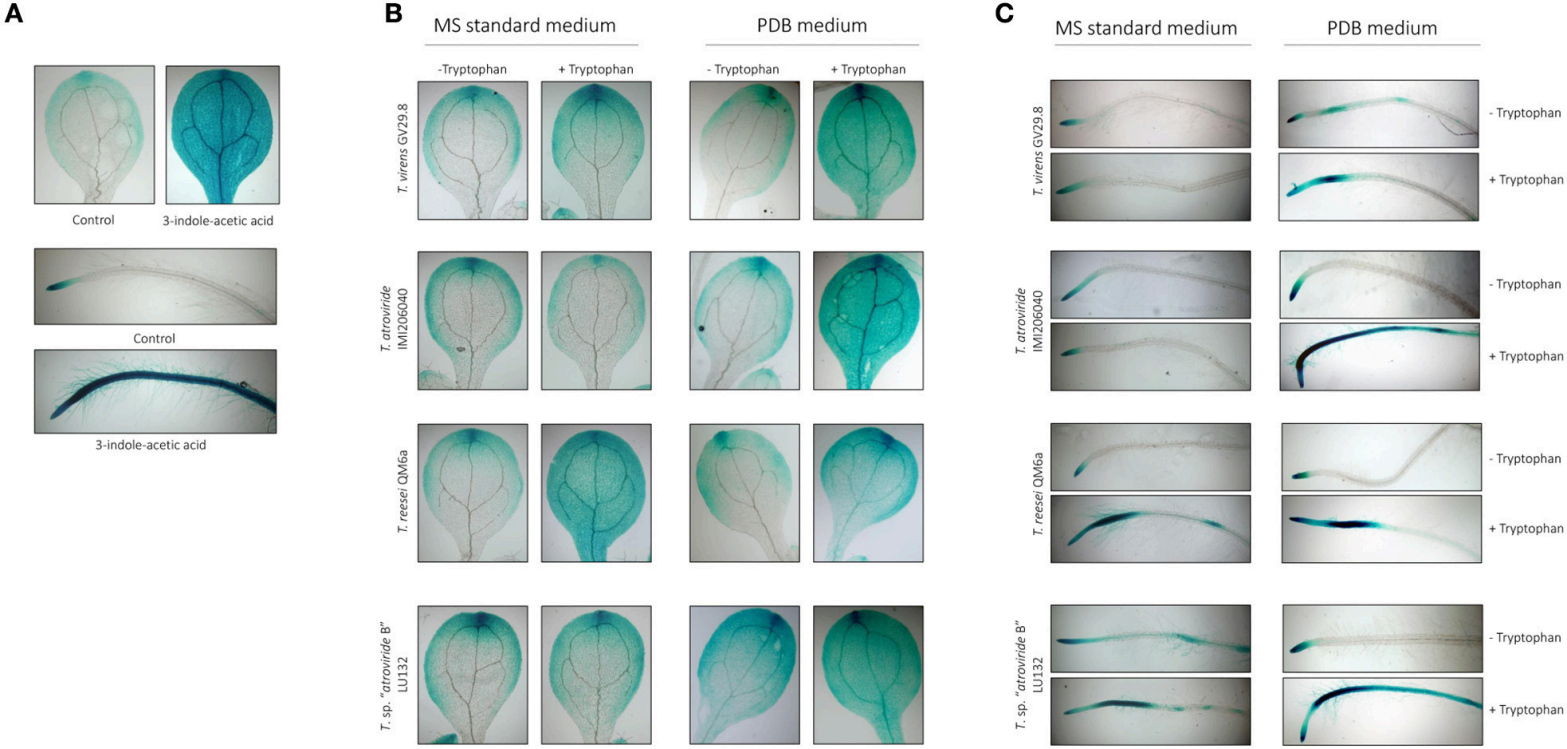
**Effect of indole-derivatives on DR5::GUS expression in 5-day-old ***A. thaliana*** seedlings. (A)** DR5::GUS expression in leaves and primary roots after treatment with ethyl acetate (control) or 1 μM indole-3-acetic acid (IAA) for 12 h. **(B)** DR5::GUS expression in leaves inoculated with 10 μL of supernatant from *Trichoderma* grown in MS supplemented or not with 10 mM Tryptophan. **(C)** DR5::GUS expression in leaves inoculated with 10 μL of supernatant from *Trichoderma* grown in PDB supplemented or not with 10 mM Tryptophan.

Strains *T*. sp. “*atroviride* B” LU132, *T. reesei* QM6a, *T. atroviride* IMI206040 and *T. virens* GV29.8 were selected for further analysis on the basis of their variable pattern on the TLC plates. Culture supernatants were assessed for their bioactivity on auxin accumulation in the reporter line *A. thaliana* DR5::GUS. *Arabidopsis* seedlings exhibited significantly more GUS activity after a 12-h treatment with 5 μM of commercial 3-indol-acetic acid than the control seedlings (Figure 6A). For the supernatants produced in MS medium, only those generated from *T. reesei* QM6a in the presence of tryptophan resulted in a clear increase in auxin accumulation in both the leaves and roots (Figures 6B,C). All culture supernatants produced in PDB in the presence of tryptophan resulted in a clear increase in auxin accumulation in both leaves and roots (Figures 6B,C).

### Growth response of *A. thaliana* to *Trichoderma* spp. volatile organic compounds (VOCs)

**Figure 7:**
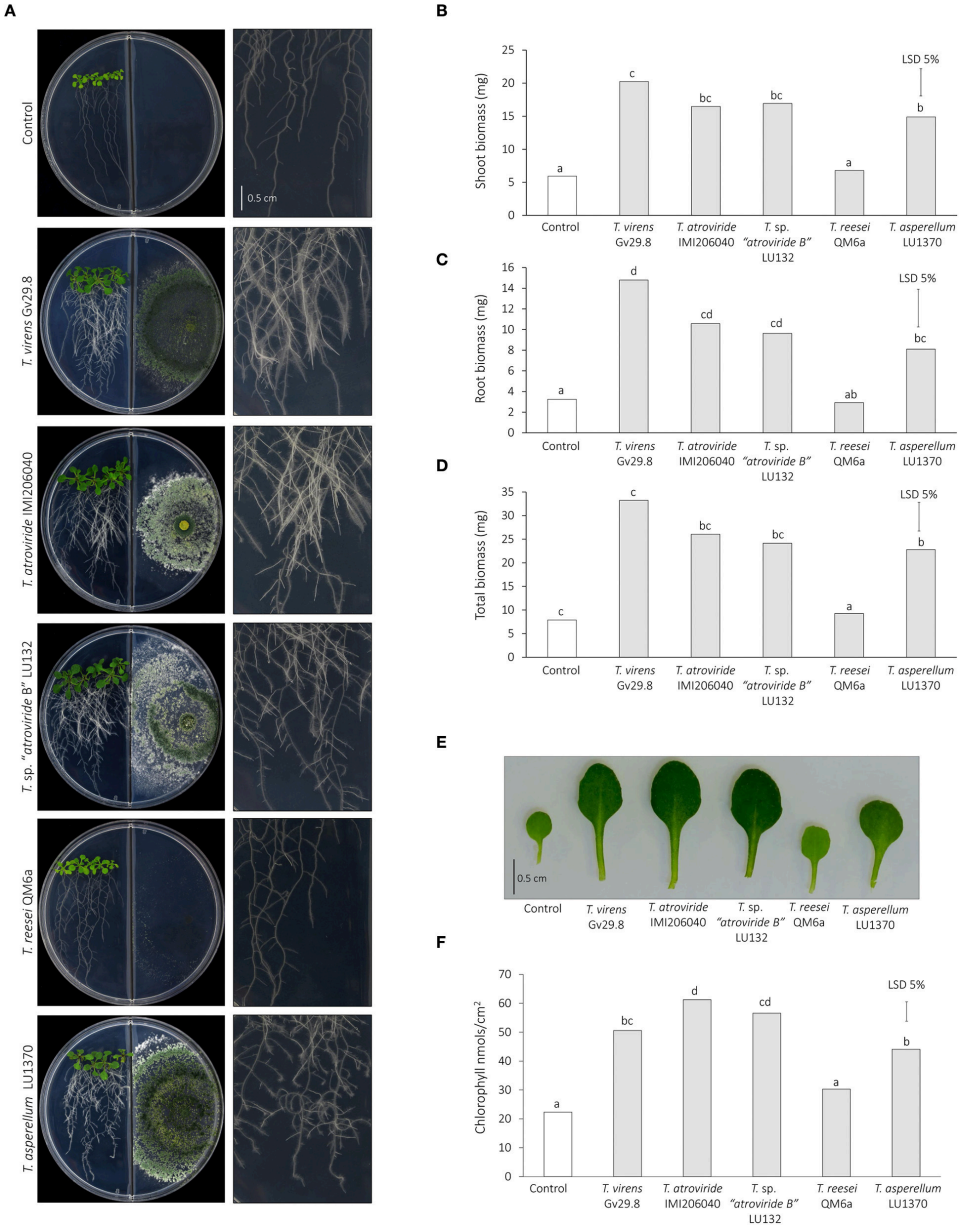
**Effect of ***Trichoderma*** volatile organic compounds on ***Arabidopsis*** plant biomass using a split-plate assay. (A)** Morphology of 14-day old seedlings co-inoculated or not with *Trichoderma* spp. in double compartment plates. A close-up of roots morphology from each interaction is illustrated in right panel. Scale bar corresponds to 0.5 mm. **(B)** Shoot biomass. **(C)** Root biomass. **(D)** Total biomass. **(E)** Magnified view of leaves exposed to *Trichoderma* spp. volatiles. Scale bar corresponds 0.5 mm. **(F)** Chlorophyll content of leaves exposed to *Trichoderma* spp. volatiles.

To assess the effect of mVOCs emitted by *Trichoderma* in growth promotion, co-cultivation experiments were done using a split-plate assay to keep both organisms physically separated. After 7 days of co-cultivation VOCs emitted by four *Trichoderma* strains (*T*. sp. “*atroviride* B” LU132 and IMI206040; *T. virens* Gv29.8 and *T. asperellum* LU1370) showed a significant increase in shoot, root and total biomass compared with the control seedlings (Figure 7). mVOCs emitted by *T. reesei* QM6a had no effect in increasing biomass in both shoots and roots with respect to the control.

Some VOCs emitted by microbes increase the efficiency of photosynthesis and chlorophyll content in *A. thaliana* by modulating the sugar content (Zhang et al., 2008). In this study, a clear difference in color was observed in leaves exposed to *Trichoderma* in comparison to the controls, with the exception of *T. reesei* QM6a which showed no difference (Figure 7E). Analysis of the chlorophyll content supported these observations; all strains except *T. reesei* QM6a induced a significant increase in chlorophyll content (Figure 7F).

### VOCs are highly variable between *Trichoderma* species

The mVOC profiles of the four strains identified as promoting growth in the absence of physical contact (section Growth Response of *A. thaliana* to *Trichoderma* spp. Volatile Organic Compounds (VOCs).) were analyzed by GCMS. *Trichoderma* sp. “*atroviride* B” LU132 and *T. atroviride* IMI206040 differed in emission of mVOCs when grown on standard MS medium; *T*. sp. “*atroviride* B” LU132 produced 6-PP in large quantities whereas *T. atroviride* IMI206040 emitted only minor quantities of this compound but higher quantities of 1-Octen-3-ol and 3-Octanone (Tables 1, 2). For *T. virens* Gv29.8, 29 sesquiterpenes were identified and of these β-Elemene and ε-Amorphene were significantly overrepresented in the mixture (Table 3). Of the mVOCs emitted by *T. asperellum* LU1370, 1,3-Octadiene, Limonene and β-Eudesmol + Valerianol were the most abundant in the mixture (Table 4).

**Table 1:** **Volatiles produced by ***Trichoderma atroviride*** IMI206040 on 0.2X standard MS medium**.

**No**.	**Class^a^**	**Compound**	**RI exp^b^**	**RI ref^c^**	**Rel. peak area**	
					**Average**	**SE**
1	C8	Octane	800	800	1.2E+05	3.5E+04
2	C8	1,3-Octadiene	824	829	2.0E+06	7.8E+05
3	Others	4-Heptanone	874	860	9.7E+04	1.2E+04
4	Others	Nonane	899	900	7.1E+04	3.0E+04
5	C8	2-Octanone	958	964	5.7E+05	3.2E+04
6	C8	(Z)-1,5-Octadien-3-ol	977	–	7.1E+05	1.1E+05
7	C8	1-Octen-3-ol	982	980	2.0E+07	6.2E+06
8	C8	3-Octanone	988	987	9.6E+06	1.3E+06
9	Others	2-Pentylfuran	993	981	8.9E+05	9.3E+04
10	Others	Unknown compund [m/z 105, 162]	1310	–	1.6E+05	1.3E+04
11	ST	α-Bergamotene	1445	1434	3.1E+05	5.7E+04
12	ST	Sesquisabinene B + β-Acoradiene	1461 + 1464	1446 + 1465	5.6E+05	2.0E+05
13	Others/ST	6-Pentyl-α-pyrone + Neocallitropsene	1471 + 1474	1469 + 1475	3.1E+06	1.9E+06
14	ST	epi-Zonarene	1492	1494	9.6E+05	2.6E+05
15	ST	β-Eudesmol	1623	1641	2.4E+05	6.8E+04
16	ST	Valerianol	1642	1647	1.3E+06	2.5E+05

a *ST (sesquiterpene), C8 (C8 compound), Others (other class of compounds)*.

b *RI exp: experimentally determined retention index*.

c *RI ref: retention index reported in literature*.

**Table 2:** **Volatiles produced by ***Trichoderma*** sp. “***atroviride*** B” LU132 on 0.2X standard MS medium**.

**No**.	**Class^a^**	**Compound**	**RI exp^b^**	**RI ref^c^**	**Rel. peak area**	
					**Average**	**SE**
1	C8	Octane	800	800	1.2E+05	1.0E+04
2	Others	2-Heptanone	891	889	1.3E+07	2.3E+05
3	Others	Nonane	899	900	1.9E+05	3.2E+04
4	C8	2-Octanone	958	964	4.5E+05	3.1E+04
5	C8	1-Octen-3-ol	982	980	3.4E+06	3.5E+05
6	C8	3-Octanone	988	987	4.9E+06	3.7E+05
7	Others	2-Pentyl furan	993	981	4.4E+06	6.7E+05
8	MT	Limonene	1032	1025	6.6E+05	2.1E+04
9	Others	2-n-Heptyl furan	1196	1196	3.7E+05	7.8E+04
10	Others	Unknown compund [m/z 105, 162]	1310	–	1.4E+05	1.0E+04
11	ST	α-Bergamotene	1445	1434	2.6E+05	5.0E+04
12	ST	Sesquisabinene B + β-Acoradiene	1461 + 1464	1446 + 1465	3.7E+05	1.0E+05
13	Others/ST	6-Pentyl-α-pyrone	1471	1469	1.1E+08	4.4E+07
14	ST	epi-Zonarene	1492	1494	1.4E+06	5.4E+05
15	ST	β-Eudesmol	1623	1641	1.4E+05	2.0E+04
16	ST	Valerianol	1642	1647	1.1E+06	7.3E+04

a *C8 (C8 compound), MT (monoterpene), ST (sesquiterpene), Others (other class of compounds)*.

b *RI exp: experimentally determined retention index*.

c *RI ref: retention index reported in literature*.

**Table 3:** **Volatiles produced by ***Trichoderma virens*** Gv29.8 on 0.2X standard MS medium**.

**No**.	**Class^a^**	**Compound**	**RI exp^b^**	**RI ref^c^**	**Rel. peak area**	
					**Average**	**SE**
1	MT	Myrcene	991	987	4.4E+06	6.9E+05
2	Ester	2-Pentenoic acid, 4,4-dimethyl-, methyl ester	1019	–	1.5E+06	1.0E+05
3	MT	Limonene	1031	1025	5.5E+05	2.9E+05
4	MT	(E)-β-Ocimene	1039	1041	2.1E+05	2.4E+04
5	Ester	Hexanoic acid, 2-ethyl-, methyl ester	1045	1043	3.0E+05	1.5E+04
6	MT	Linalool	1102	1086	3.6E+05	2.8E+04
7	ST	Bicycloelemene	1346	1338	7.7E+05	2.0E+05
8	ST	β-Cubebene	1390	1390	8.7E+05	1.7E+05
9	ST	Cis-β-Elemene	1395	1381	3.7E+06	8.4E+05
10	ST	β-Elemene	1403	1389	5.0E+07	1.1E+07
11	ST	α-Gurjunene	1424	1419	2.2E+06	4.6E+05
12	ST	Tritomarene	1429	1416	9.8E+05	2.0E+05
13	ST	(E)-β-Caryophyllene	1434	1421	1.4E+07	2.8E+06
14	ST	Isogermacrene D	1443	1445	8.6E+05	1.5E+05
15	ST	4aH,10aH-Guaia-1(5),6-diene	1448	1445	9.0E+05	1.2E+05
16	ST	Aromadendr-9-ene	1457	1463	1.8E+06	3.6E+05
17	ST	Carota-5,8-diene	1465	1465	1.7E+06	3.9E+05
18	ST	α-Humulene	1470	1455	1.3E+06	1.2E+05
19	ST	Germacrene D	1479	1479	2.2E+06	1.1E+05
20	ST	epi-Zonarene	1488	1494	1.1E+07	1.2E+06
21	ST	ε-Amorphene	1497	1498	3.0E+07	5.1E+06
22	ST	Isogermacrene A	1504	1502	7.6E+06	1.7E+06
23	ST	Guaia-1(10),11-diene	1513	1516	1.9E+07	3.0E+06
24	ST	Guaia-9,11-diene	1522	1522	4.8E+06	3.2E+05
25	ST	Unknown sesquiterpene	1530	–	9.5E+06	4.7E+05
26	ST	ω-Amorphene	1538	1526	1.2E+07	4.4E+06
27	ST	Selina-3,7(11)-diene	1541	1542	3.9E+06	5.7E+05
28	ST	Unknown sesquiterpene	1547	–	1.1E+06	2.1E+05
29	ST	Unknown sesquiterpene	1553	–	6.0E+05	5.5E+04
30	ST	γ-Calacorene	1560	1554	5.5E+05	7.1E+04
31	ST	Palustrol	1589	1569	1.4E+06	2.1E+05
32	ST	Ledol	1613	1600	2.0E+07	2.5E+06
33	ST	τ-Muurulol	1665	1633	2.3E+06	2.1E+05
34	ST	Unknown sesquiterpene	1677	–	3.2E+05	1.4E+05
35	ST	γ-1-Cadinene aldehyde	1796	–	1.4E+05	9.1E+03

a *MT (monoterpene), ST (sesquiterpene)*.

b *RI exp: experimentally determined retention index*.

c *RI ref: retention index reported in literature*.

**Table 4:** **Volatiles produced by ***Trichoderma asperellum*** LU1370 on 0.2X standard MS medium**.

**No**.	**Class^a^**	**Compound**	**RI exp^b^**	**RI ref^c^**	**Rel. peak area**	
					**Average**	**SE**
1	C8	Octane	800	800	9.5E+04	4.9E+03
2	C8	1,3-Octadiene	825	829	2.1E+06	3.3E+05
3	Others	Nonane	899	900	6.5E+04	1.1E+04
4	Others	2-Pentyl furan	993	981	6.7E+05	6.8E+04
5	MT	Limonene	1032	1025	9.8E+05	1.3E+05
6	Others	Unknown compund [m/z 105, 162]	1310	–	4.7E+05	1.2E+04
8	ST	β-Acoradiene	1464	1465	2.3E+05	6.9E+04
9	ST	Neocallitropsene	1471	1469	3.6E+05	1.1E+05
10	ST	epi-Zonarene	1492	1494	6.1E+05	7.1E+04
11	ST	β-Eudesmol + Valerianol	1642	1647	1.4E+06	1.4E+05
12	DT	Isopimara-8, 15-diene	1924	1922	2.5E+05	1.8E+04
13	DT	Unknown diterpene	1931	–	4.4E+05	1.7E+04
14	DT	Unknown diterpene	1972	–	1.4E+05	1.7E+04

a *MT (monoterpene),ST (sesquiterpene), DT (diterpene)*.

b *RI exp: experimentally determined retention index*.

c *RI ref: retention index reported in literature*.

## Discussion

### Plant growth promotion is not a universal trait of all *Trichoderma* strains

In nature, multiple symbiotic relationships form between plants and microorganisms and these relationships can be manipulated to improve plant productivity. One such trait is plant growth promotion. It has been reported that several species of *Trichoderma* have the ability to induce growth promotion on diverse crop plants regardless of the place from which they were isolated (Harman et al., 2004; Mastouri et al., 2010; Lee et al., 2016). The *Trichoderma* strains tested in this study were collected from both free-living and plant symbiotic sources and, as has been reported, there appeared to be no correlation between isolation source and the ability to promote plant growth. However, our work clearly demonstrates that growth promotion is not a universal trait of all *Trichoderma*. Similar to that observed by Lee et al. (2016), we observed promotion, no effect at all, and detrimental effects on plants. Further, the variability in plant growth promoting potential appears to be more strongly influenced by environmental parameters than the choice of plant host. This work highlights the need for greater research into the environmental control of successful plant health enhancement.

Detailed biochemical studies on growth promotion, by necessity, require the use of *in vitro* systems, however the choice of system can directly affect the outcome and will not necessarily reflect how the microorganism behaves in nature. Originally isolated from healthy *Hydrangea* roots, when tested in sterile soil in this study, *T. asperellum* LU1370 significantly reduced *Arabidopsis* fresh weight. Interestingly, this strain also significantly reduced the survival of *Lophomyrtus* cuttings by 40% (data not shown) and had no effect on the growth parameters of tomato seedlings (M Braithwaite, unpublished data.). However, when *T. asperellum* LU1370 was tested on agar plates, a positive effect on *Arabidopsis* growth was observed.

The inhibitory effects of *Trichoderma* on plants have previously been reported, particularly with respect to seed germination on diverse plants including lettuce, onion and chicory (Ousley et al., 1993; Celar and Valic, 2005). *Trichoderma* spp. synthesize a plethora of secondary metabolites with diverse biological function in plants (Reino et al., 2008). For example, trichosetin, a secondary metabolite isolated from dual cultures of *T. harzianum-Catharanthus roseus* callus (Marfori et al., 2002), inhibited root and shoot growth in five plant species (*Oryza sativa, Vigna radiate, Medicago sativa, Capsicum frutescens*, and *Lycopersicon esculentum*) by damaging the cell membrane (Marfori et al., 2003). Roots treated with trichosetin were mostly dead, indicating a clear phytotoxic effect of this compound (Marfori et al., 2003). Additional compounds with negative effects on plant growth include Trichocaranes (A, B, and C) (Macías et al., 2000), konionginins (B, C, E, and G) (Cutler et al., 1989; Parker et al., 1995), cyclonerodiol (Cutler et al., 1991), and a laevorotatory form of harzianopyridone (Cutler and Jacyno, 1991). The last compound caused necrosis in bean, tobacco and corn in a concentration dependent manner (Cutler and Jacyno, 1991). *Trichoderma virens* also synthesizes negative plant growth promoters such as viridiol, a potent herbicidal compound, which is effective for weed control (Héraux et al., 2005).

The biosynthesis of secondary metabolites is modulated by environmental conditions. The general effect of *T. asperellum* LU1370 on plant growth in soil was an inhibition in plant size without causing plant death, by contrast, on plate assays the general effect was stimulatory, suggesting that either different molecules are produced in both environments (soil and plates) or the proportion of specific metabolites are differently accumulated in these conditions. The dual effect of specific metabolites have been documented before, for example, 6-pentyl- 2H-pyran-2-one (6-PP) induces plant growth promotion, depending on the concentration (Garnica-Vergara et al., 2015). At high concentrations 6-PP has an inhibitory effect and at low concentrations it promoted growth in wheat seedlings (Vinale et al., 2008). However, Lee et al. (2016) recently reported no correlation between 6-PP and growth promotion. *Trichoderma asperellum* IsmT5 synthesizes 6-PP (Kottb et al., 2015) but we did not identify this molecule in *T. asperellum* LU1370, although it may be induced by the plant as was recently reported for *T. atroviride* (Garnica-Vergara et al., 2015). The role of 6-PP in the strains presented in this study is unknown.

### Production of indole-3 acetic acid by *Trichoderma* is determined by environmental conditions

It is known that some microorganisms have the ability to produce phytohormones like IAA (Robinson et al., 1998; Contreras-Cornejo et al., 2011; Hilbert et al., 2012; Fu et al., 2015).

In bacteria, indole acetic acid (IAA) biosynthesis varies significantly depending on the media used (Spaepen et al., 2007). In fungi, the most well-known mechanism of IAA biosynthesis is tryptophan-dependent, and consequently the addition of this amino acid to the media results in the production of higher levels of IAA (Robinson et al., 1998; Chung et al., 2003; Spaepen and Vanderleyden, 2011; Hilbert et al., 2012). An example of how media determine the production of IAA is shown in Figure 5 where *T. virens* and *T. atroviride* synthesize IAA in PDB supplemented with L-Tryptophan but not in MS supplemented with the same amino acid. The ambient pH was different between the PDB and MS media, pH 5.8 ± 0.2 and pH 7.0 respectively. MS media used in this study contained 0.6% sucrose while PDB formulation contained 2% glucose (dextrose), these differences may have an impact on the mechanisms of synthesis of IAA. In fungi, environmental factors, including pH and temperature have an impact on IAA biosynthesis (Yu et al., 1970; Strzelczyk et al., 1992; Bose et al., 2013; Sun et al., 2014). Carbon and nitrogen sources have been shown to be essential factors influencing fungal and bacterial IAA biosynthesis (Yurekli et al., 2003; Shokri and Emtiazi, 2010); whether they play an intrinsic role in fungal IAA production remains to be explored.

By comparing MS medium supplemented with or without L-tryptophan, Salas-Marina et al. (2011) reported a ~2-fold increase of IAA derivatives secreted by *T. atroviride* IMI206040 and *T. virens* Gv298. This does not contradict to findings because the authors reported a total amount of indole derivatives using the Salkowski's reagent, which is a colorimetric method to detect general indole substances (Perley and Stowe, 1966). In this study, *T. atroviride* IMI206040 secreted multiple indole derivatives, however, the Rf differed from the commercial IAA standard. Furthermore, the indole derivatives induced in MS from *T. atroviride* IMI206040 and *T. virens* Gv29.8 did not display auxin activity. Further experimental work is required to understand the mechanisms of IAA synthesis by *Trichoderma* and how this molecule impacts plant physiology.

### Auxin production does not strictly correlate with plant growth promotion

Currently IAA is thought to be a diffusible signal involved in interspecies communication (Fu et al., 2015); however, the final outcome of this communication is dependent on the organisms interacting and is also influenced by the environment where the interaction occurs. For example, while plant growth promotion induced by *T. virens* Gv29.8 and *T. atroviride* IMI206040 has been related to their ability to secrete IAA (Contreras-Cornejo et al., 2009), some other studies have observed no relation between IAA production and growth induction, at least when plants are tested in soil. Hoyos-Carvajal et al. (2009a) identified that of 101 *Trichoderma* strains collected from Colombia, 60% produced IAA-derivatives *in vitro* but only 18% had the ability to induce plant growth promotion in beans. In the present study, some *Trichoderma* strains produced significant quantities of IAA *in vitro* and also increased *Arabidopsis* seedling shoot fresh weight in soil experiments, providing a possible link between the two systems. However, the link between IAA production and plant growth promotion was not consistent for all strains. For instance, in the *in vitro* agar plate tests, five of the *Trichoderma* strains were similar to *T. atroviride* IMI206040 and *T. virens* Gv29.8 with respect to increases in lateral root growth and root density, but not all isolates produce IAA in liquid MS supplemented with L-Trp. A similar lack of correlation between growth promotion and 6-PP production was recently observed in *Trichodema* (Lee et al., 2016).

Several authors have reported that bacteria, microalgae, fungi, and plants exchange IAA as a signaling molecule that affects their physiology (Fu et al., 2015). The rice blast fungus *Magnaporthe oryzae* synthesizes IAA specifically in the area of the infection hyphae (Tanaka et al., 2011), but it is not clear whether this occurs to manipulate the host plant or to benefit its own self. Furthermore the endophytic fungus *Piriformospora indica* synthesizes IAA not for growth promotion but for plant colonization (Hilbert et al., 2012), suggesting that additional mechanisms must exist for this process. Further molecular studies on *Trichoderma* are required to understand the mechanisms of synthesis in the fungus and how this molecule affect the interaction with plant roots.

Altogether, our findings suggest that IAA production seems not to be a key determinant for plant growth promotion. However, in this stage we cannot discard the possibility that plants are triggering IAA production in *Trichoderma* as occurs in other microorganisms including *Xanthomonas axonopodis* (Costacurta et al., 1998) and *Magnaporthe oryzae* (Fu et al., 2015).

### *Trichoderma* directly affects auxin localisation within *Arabidopsis*

Cell polarity has been crucial for evolution of multicellular organisms (Offringa and Kleine-Vehn, 2013) and is one of the fundamental aspects of development (Dhonukshe, 2009). In plants, PIN proteins are integral membrane proteins implicated in the directional efflux of IAA (Luschnig and Vert, 2014). These proteins can alter their localization from one cell to another by developmental and environmental conditions (Friml et al., 2002, 2003, 2004; Kleine-Vehn et al., 2008). Our results showed that *Trichoderma* spp. inhibit primary root elongation and increase lateral root numbers (This study; Salazar-Badillo et al., 2015). Our results showed that in the primary tip, *Trichoderma* promotes a reduction in the accumulation of DR5::GUS and DR5::GFP expression, indicating a reduction of DR5 activity (Figures 3, 4). When the *T. virens* Gv29.8 and *Arabidopsis* interaction was analyzed, we observed decreased expression or delocalization of auxin PIN receptors. This reduction in the accumulation of PIN receptors might be the cause of the reduction of *A. thaliana* root length in the interaction with *Trichoderma*. Recently it was reported that Trichokonin VI (TK VI), a peptaibol produced by *T. longibrachiatum* SMF2, inhibits primary root growth on *Arabidopsis* by suppressing cell division and cell elongation, and disrupting root stem cell niche maintenance (Shi et al., 2016). Contreras-Cornejo et al. (2015) recently reported that the mitogen-activated protein kinase 6 (MPK6) has an important role in the induction of root length during the interaction with *T. atroviride*. Mutants of the mpk6 have an enhanced root inhibition when interacting with *Trichoderma*. The fact that *Trichoderma* spp. strongly activates DR5 in the secondary roots also suggests that additional molecules that work at distance might be involved, such as volatile organic compounds emitted by the fungi as was recently reported in *T. atroviride* (Garnica-Vergara et al., 2015).

### *Trichoderma* volatile organic compounds (VOCs) actively promote plant growth

Microbial volatile organic compounds have been described as important inducers of growth promotion in bacteria and fungi, including mycorrhizae and *Trichoderma* (Garnica-Vergara et al., 2015). The mechanisms behind microbial-induced plant growth promotion are complex, and, in the case of the strains discussed in the present work, may involve IAA but not as a key determinant. Although their exact mechanism is not known, the results from this study clearly demonstrate that VOCs are able to actively promote plant growth in the absence of physical fungus-plant contact. Four out of five strains tested in this study were able to increase plant biomass and chlorophyll content when physically separated from the plant. However, the wide and varied array of VOCs produced by the various strains suggests that non-contact induction may be due to a number of diverse compounds and mechanisms. Garnica-Vergara et al. (2015) reported that 6-pentyl-2H-pyran-2-one (6-PP) from *T. atroviride* is a key element in growth promotion. Interestingly, Garnica-Vergara et al. (2015) observed that higher concentrations of the pyrone induce a reduction of the primary root length. However, the concentrations used can be far from those observed in natural conditions. Furthermore, Lee et al. (2016) reported no correlation between 6-PP production and growth promotion.

In our study, we observed an atonishing diversity of VOCs produced by different strains, suggesting the existence of diverse mechanisms of induction. However, we cannot exclude the possibility of and influence of CO_2_ in plant growth promotion as has been suggested before (Ramadan et al., 2015), although Garnica-Vergara et al. (2015), and Kottb et al. (2015) discarded this possibility by using pure compounds instead of the fungi. In addition, Lee et al. (2016) recently reported that the amount of CO_2_ generated by *Trichoderma* spp. is insufficient to induce *Arabidopsis* growth promotion and the effects observed are attributable to VOCs synthesized by *Trichoderma* spp.

In summary, this study provides evidence that the communication between plants and *Trichoderma* involves the recognition of fungal-derived molecules such us auxins and VOCs, however this communication is strongly dependent on the environment. Many studies have been conducted on the effect of *Trichoderma* metabolites. However, there is a lack of strong evidence of a correlation between growth promotion and the production of a particular compound, suggesting a complex interplay of molecules is required. Future molecular studies examining the roles of specific genes and pathways may shed further light on the role of fungal-derived molecules in promotion of plant growth.

## Author contributions

MFNJ, FBSB, DVN, MR, MB, JMS, MO, and AMM performed experimental work; MFNJ, AMM, JFJB, MB, and MR designed the experiments; MFNJ, AMM, FBSB, JFJB, MB, and JMS discussed and interpreted the results; AMM and AS designed the research, contributed to chemicals, and scientific advice; MFNJ, JMS, and AMM wrote the paper. All authors reviewed the final version of the paper.

## Funding

This work was supported by the Pre-Seed Accelerator Fund (PSAF), the Tertiary Education Commission (contracts 38631 and 38651) and the Ministry for Science and Innovation. Fatima Salazar was supported by Tertiary Education Commission and Conacyt-Mexico for her stay at Lincoln University. JT acknowledges the financial support of CNPq and CAPES.

### Conflict of interest statement

The authors declare that the research was conducted in the absence of any commercial or financial relationships that could be construed as a potential conflict of interest.
