# Supplementary material for: Environmental Growth Conditions of Trichoderma spp. Affects Indole Acetic Acid Derivatives, Volatile Organic Compounds, and Plant Growth Promotion
Source: Front Plant Sci. 2017 Feb 9;8:102. doi: 10.3389/fpls.2017.00102 (PMC5299017; doi:10.3389/fpls.2017.00102)
Supplement: Supplementary file 1 [file DataSheet1.docx]

Supplementary Material

Multiple Signals Derived from *Trichoderma* spp. Control Plant Fitness^&^

Maria Fernanda Nieto-Jacobo^1^, Johanna M Steyaert^1^, Fatima Berenice Salazar-Badillo^1,2^, Dianne Nguyen^1^, Michael Rostás^1^, Mark Braithwaite^1^, Jorge Teodoro de Souza^1,3^, Juan Francisco Jimenez-Bremont^2^, Mana Ohkura^4^, Alison Stewart^5^ and Artemio Mendoza Mendoza^1*^

^1^Bio-Protection Research Centre, Lincoln University, Lincoln, New Zealand

^2^Institute for Scientific and Technological Research of San Luis Potosi, San Luis Potosí, México

^3^ Department of Phytopathology, Federal University of Lavras, Lavras, MG, Brazil

^4^ School of Plant Sciences, University of Arizona, Tucson, AZ, USA

^5^ SCION, Rotorua, New Zealand

*** Correspondence**Artemio Mendoza Mendoza
[artemio.Mendoza@lincoln.ac.nz](mailto:artemio.Mendoza@lincoln.ac.nz)

**Supplementary Figure 1S.** Anthocyanin induction in *A. thaliana* after interaction with different *Trichoderma* spp. Olympus BX51 compound microscope was used and images were captured using an Olympus DP70 digital camera system and processed with the software CellF (Olympus).


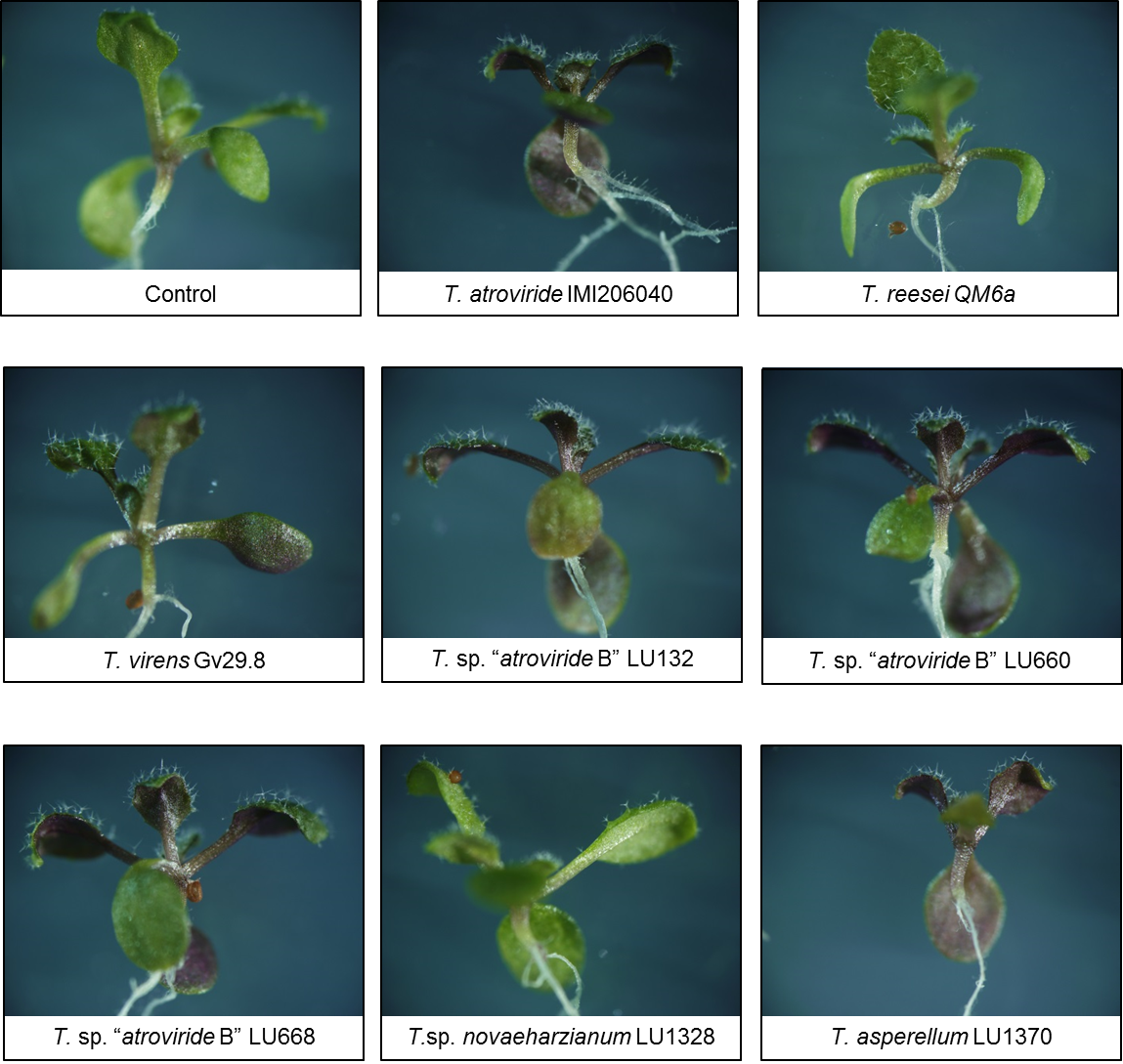


| **Supplementary Table 1S:** *Trichoderma strains* used in this study | | | |
| --- | --- | --- | --- |
| **Species** | **Strain** | **Habitat** | **Geographic location, date isolation** |
| *Trichoderma trixiae* | LU297 | *Vitis* sp. | Auckland, New Zealand, 2000 |
| *Trichoderma* sp. *"atroviride*  B*"* | LU660 | *Salix sp.* | New Zealand, 1990 |
| *Trichoderma* sp. *"atroviride*  B*"* | LU668 | Soil | Te Puna, New Zealand |
| *Trichoderma sp. nov* | LU753 | Soil under grass, Lincoln University, New Zealand | Canterbury, New Zealand, 2002 |
| *Trichoderma sp. "novaeharzianum"* | LU1328 | *Carex* sp. Roots | Canterbury, New Zealand, 2009 |
| *Trichoderma asperellum* | LU1370 | *Hydrangea* roots | Canterbury, New Zealand, 2009 |
| *Trichoderma* sp. *"atroviride*  B*"* | LU132 | Soil under onion crop | Pukekoe, New Zealand, 2000 |
| *Trichoderma atroviride* | IMI206040 | *Picea excelsa* (plum tree) | Sweden, 1976 |
| *Trichoderma virens* | Gv29-8 | Agricultural soil near College Station, Texas | Texas, USA, 1992 |
| *Trichoderma reesei* | QM6a | Cotton duck shelter | Solomon Islands, Bougainvillea Island, World War II |

| **Supplementary Table 2S:** List of the *Arabidopsis* lines used in this study | | | |
| --- | --- | --- | --- |
| ***Arabidopsis thaliana* Ecotype** | **Genotype** | **Tagged protein** | **References** |
| *Arabidopsis thaliana* | *A. thaliana* Col-0 Wild type |  |  |
| *Arabidopsis thaliana* Col0 | *A. thaliana* Col-0 DR5::GUS | Synthetic auxin response element fused to beta-glucuronidase encoding gene | Ulmasov *et al*. 1997. |
| *Arabidopsis thaliana* Col0 | *A. thaliana* Col-0 DR5rev::GFP | Synthetic auxin response element fused to green fluorescent protein encoding gene | Friml *et al.* 2003 |
| *Arabidopsis thaliana* Col0 | *A. thaliana* Col-0 PIN1::PIN1-GFP | *A. thaliana* auxin efflux carrier Pin-Formed 1 (AT1G73590) | Benkova *et al.* 2003 |
| *Arabidopsis thaliana* Col0 | *A. thaliana* Col-0 PIN2::PIN2-GFP | *A. thaliana* auxin efflux carrier Pin-Formed 2 (AT5G57090) | Xu and  Scheres 2005 |
| *Arabidopsis thaliana* Col0 | *A. thaliana* Col-0 PIN4::PIN4-GFP | *A. thaliana* auxin efflux carrier Pin-Formed 4 (AT2G01420) | Blilou *et al.* 2005 |
| *Arabidopsis thaliana* Col0 | *A. thaliana* Col-0 PIN7::PIN7-GFP | *A. thaliana* auxin efflux carrier Pin-Formed 7 (AT1G23080) | Blilou *et al*. 2005 |
| *Arabidopsis thaliana* Col0 | *A. thaliana* Col-0 PIN3::PIN3-GFP | *A. thaliana* auxin efflux carrier Pin-Formed 3 (AT1G70940) | Zadnikova *et al.* 2010 |
